# Altered neural response to rejection‐related words in children exposed to maltreatment

**DOI:** 10.1111/jcpp.12595

**Published:** 2016-07-26

**Authors:** Vanessa B. Puetz, Essi Viding, Amy Palmer, Philip A. Kelly, Rachael Lickley, Iakovina Koutoufa, Catherine L. Sebastian, Eamon J. McCrory

**Affiliations:** ^1^ Division of Psychology and Language SciencesUniversity College LondonLondonUK; ^2^ Anna Freud CentreLondonUK; ^3^ Department of PsychologyRoyal HollowayUniversity of LondonEghamUK

**Keywords:** Child abuse, emotion regulation, fMRI, posttraumatic stress disorder, adolescence

## Abstract

**Background:**

Children exposed to maltreatment show neural sensitivity to facial cues signalling threat. However, little is known about how maltreatment influences the processing of social threat cues more broadly, and whether atypical processing of social threat cues relates to psychiatric risk.

**Methods:**

Forty‐one 10‐ to 14‐year‐old children underwent a social rejection‐themed emotional Stroop task during functional magnetic resonance imaging: 21 children with a documented history of maltreatment (11 F) and 19 comparison children with no maltreatment history (11 F). Groups were matched on age, pubertal status, gender, IQ, socioeconomic status, ethnicity and reading ability. Classic colour Stroop stimuli were also administered in the same paradigm to investigate potential differences in general cognitive control.

**Results:**

Compared with their peers, children who had experienced maltreatment showed reduced activation in the Rejection versus Neutral condition, across circuitry previously implicated in abuse‐related posttraumatic stress disorder (PTSD), including the left anterior insula, extending into left ventrolateral prefrontal cortex/orbitofrontal cortex; left amygdala; left inferior parietal cortex (STS); and bilateral visual association cortex, encompassing the cuneus and lingual gyrus. No group differences in neural or behavioural responses were found for the classic colour Stroop conditions. Significant negative associations between activity in bilateral cuneus and STS during the rejection‐themed Stroop and higher self‐reported PTSD symptomatology, including dissociation, were observed in children exposed to maltreatment.

**Conclusion:**

Our findings indicate a pattern of altered neural response to social rejection cues in maltreated children. Compared to their peers, these children displayed relative hypoactivation to rejection cues in regions previously associated with PTSD, potentially reflecting an avoidant coping response. It is suggested that such atypical processing of social threat may index latent vulnerability to future psychopathology in general and PTSD in particular.

## Introduction

Childhood maltreatment, including neglect, is associated with a wide range of maladaptive outcomes for mental and physical health as well as social functioning (Lansford et al., 2002). Maltreatment significantly increases risk for psychiatric disorders, including posttraumatic stress disorder (PTSD) and depression (Vachon, Krueger, Rogosch, & Cicchetti, 2015). The theory of latent vulnerability (McCrory & Viding, 2015) provides one framework within which to conceptualise the association between maltreatment and psychopathology. It contends that there are calibrations in biological and neurocognitive systems in response to early risk environments; while adaptive in the short term, these can confer long‐term risk for psychiatric disorders following future stressors (McCrory & Viding, 2015). Such changes to neurocognitive systems should be measurable in childhood, allowing the identification of psychiatric risk mechanisms in the absence of overt symptomatology (Hanson, Hariri, & Williamson, 2015).

The processing of threat‐related cues represents one candidate neurobiological mechanism susceptible to stress‐induced alteration (McCrory & Viding, 2015). Maltreatment experience has been associated with heightened perceptual salience of negative stimuli, specifically threatening (i.e. angry) facial expressions (McCrory et al., 2011; Pollak, Vardi, Bechner, & Curtin, 2005). Several studies have demonstrated that maltreated children show an enhanced response to threatening facial expressions at the behavioural (Pollak et al., 2005) and neural levels, with altered functioning reported in the amygdala, anterior insula and prefrontal cortices (e.g. van Harmelen et al., 2014; McCrory et al., 2011). These structures have also been implicated in the psychopathology of affective disorders (Etkin & Wager, 2007) commonly elevated in individuals with maltreatment histories.

Two recent fMRI studies have aimed to assess the processing of more socially complex constructs in maltreated children. Using a Cyberball paradigm, which simulates the experience of social rejection, these studies have demonstrated that maltreatment experience is associated with heightened distress and altered neural activity to social rejection (van Harmelen et al., 2014; Puetz et al., 2014). The experience of social rejection is an established risk factor for psychopathology and poor academic performance in the population at large (Masten et al., 2009; Platt, Cohen Kadosh, & Lau, 2013; Sebastian, Viding, Williams, & Blakemore, 2010; Silk et al., 2014). Children who have experienced maltreatment are at higher risk for being rejected by their peers from childhood to adolescence (Bolger & Patterson, 2001) and show qualitative differences in interpersonal relationships into adulthood (Wolfe, Scott, Wekerle, & Pittman, 2001). This work is consistent with the finding that adults with childhood histories of maltreatment present with negative cognitive self‐schemas and biases (van Harmelen et al., 2010; Zeanah & Zeanah, 1989), which in turn may moderate the effect of social rejection on the development of affective disorders (O'Dougherty Wright, Crawford, & Del Castillo, 2009; Shields & Cicchetti, 2001). One possibility is that maltreatment leads to altered salience of social threat cues, with implications for attentional allocation and emotional and behavioural regulation. This could in turn influence the way in which these individuals interact with others and are perceived by peers.

The current study aimed to investigate neural responses to rejection‐themed words in a group of children with documented experiences of maltreatment. Specifically, we explored whether such words would be associated with heightened affective interference on cognitive control processes during an emotional Stroop (ES) task (see Williams, Mathews, & MacLeod, 1996). ES is a modified version of the classic colour‐naming Stroop (Stroop, 1935), where interference during colour‐naming of emotionally valenced words is thought to indicate attentional biases in response to affective information (see De Ruiter & Brosschot, 1994 for a review). We employed an established version of this paradigm previously used in typical and clinical adolescent and adult populations, where the affective information consists of negative self‐relevant information that is rejection‐themed words (Chechko et al., 2013; Sebastian, Roiser, et al., 2010). While evidence of affective interference at the behavioural level has been mixed (Dalgleish et al., 2003), fMRI studies have been relatively consistent in demonstrating an association between such interference and a network of emotion processing and regulatory regions including the ventromedial and ventrolateral prefrontal cortex (vmPFC/vlPFC); specifically the inferior frontal gyrus (IFG), the anterior cingulate cortex (ACC) the amygdala, the insula as well as the visual association cortex/cuneus (Chechko et al., 2013; Sebastian, Roiser, et al., 2010). Altered activation of these same areas (patterns of increased and decreased activation, depending on the population and the task) has been implicated in ES paradigms in patients with PTSD (Bremner et al., 2004; Thomaes et al., 2012), depression (Chechko et al., 2013) and anxiety disorders (Dresler et al., 2012). Reduced activation in emotion processing and regulatory areas may reflect a pattern of functional avoidance; such a pattern has been reported for PTSD patients (for whom avoidance is a core feature), during ES tasks.

‘Hybrid versions’ of the task in which a classic colour condition (i.e. nonvalenced incongruent colour words such as red written in green ink) is implemented alongside the emotionally valenced conditions, enables the investigation of group differences in interference that are specific to affective valence as well as those that are primarily related to differences in cognitive control (Bremner et al., 2004; Chechko et al., 2013; Thomaes et al., 2012). The use of a hybrid version is especially important in the present study, as previous studies have demonstrated mixed evidence regarding deficits in executive function and cognitive control in maltreated samples (e.g. Kirke‐Smith, Henry, & Messer, 2014).

Using a hybrid Stroop task comprising both rejection‐themed words as well as classic incongruent colour words, we predicted group differences in rejection‐themed words in maltreated compared with nonmaltreated children in regions previously showing atypical activation during affective interference in PTSD and depression (i.e. vmPFC, vlPFC/IFG, ACC, insula, visual association cortices and amygdala; Bremner et al., 2004; Chechko et al., 2013; Thomaes et al., 2012). We did not make directional predictions in relation to either decreased (possibly reflecting avoidance/more shallow processing) or increased (possibly reflecting hypervigilance) neural activity in this circuit, for two reasons. First, this circuit has been reported to show both atypical increases and decreases in neural activity during affective interference tasks in clinical samples with PTSD and depression, both conditions associated with maltreatment experience. Second, affective interference during a cognitively demanding task such as the Stroop differs from the low cognitive demands of previous studies investigating threat processing in maltreated children and therefore it is difficult to use these prior studies to inform clear directional predictions (McCrory et al., 2011; McCrory et al., 2013).

The amygdala was examined as a region of interest (ROI) given its established involvement in threat processing in general (Phelps & LeDoux, 2005) and in processing rejection‐themed words specifically (Sebastian, Roiser, et al., 2010). Given the evidence of altered processing in these regions during affective interference in PTSD and depression, we conducted correlational analyses between symptomatology across these domains and neural response in the Maltreated group. Finally, in view of the limited evidence regarding executive processing deficits in children with maltreatment experience, the classic colour Stroop condition was regarded as exploratory.

## Methods

### Participants

A total of forty 10‐ to 14‐year olds were recruited for this study. Twenty‐one children with a documented experience of maltreatment (mean age = 12.47 *±* 1.66 years; *N *=* *11 female) were recruited from a London Social Services (SS) Department. Information on the nature, severity and duration of maltreatment was obtained through independent ratings by the child's social worker (*N *=* *16) or adoptive parent (*N *=* *5). An additional 19 nonmaltreated children were recruited from primary and secondary schools, after‐school youth clubs in the London area, and via newspaper and Internet advertisement. Exclusion criteria for the Nonmaltreated group included any previous contact with SS with regard to the quality of parental care or maltreatment. Participants across groups were comparable in age, pubertal status, sex, handedness, IQ, reading ability, socioeconomic status (income, level of education and employment status all *p*s *> *.17) and ethnicity (see Table 1). Consent was obtained from the child's legal guardian. Assent to participate in the study was obtained from all children. Exclusion criteria for all participants included a diagnosis of learning disability, pervasive developmental disorder, neurological abnormalities, standard MRI contraindications (e.g. ferromagnetic implants) and IQ < 70. All procedures in the study were approved by University College London Research Ethics Committee (0895/002).

**Table 1:** Demographic and background information for Maltreated and Nonmaltreated groups

Measure	Maltreated group (*n* = 21)	Nonmaltreated Group (*n* = 19)	*p*
	Mean (*SD*)	Mean (*SD*)	
Age (years)	12.47 (1.66)	12.91 (1.32)	.37
WASI‐IQ^a^	105.24 (15.80)	106.21 (12.36)	.83
Reading score (WRAT^a^)	112.95 (20.07)	116.45 (15.02)	.54
Verbal fluency	35 (11.54)	36.78 (5.93)	.54
Pubertal development (PDS)^b^	2.06 (0.81)	1.84 (0.47)	.36

	*n* (%)	*n* (%)	*p*
Gender (% female)	11 (52)	11 (58)	.73
Ethnicity (% Caucasian)	15 (71)	12 (63)	.58
SES^c^	2.74 (1.88)	3 (1.28)	.15

	Mean (*SD*)	Mean (*SD*)	*p*
CTQ^d^ (total)	36.74 (15.90)	28.29 (4.99)	.02
TSCC^e^			
Anxiety	44.91 (8.12)	42.11 (7.64)	.27
Depression	45.10 (6.59)	40.58 (6.50)	.04
Anger	45.14 (11.32)	39.63 (5.95)	.07
PTSD	43.38 (5.55)	40.79 (6.03)	.17
Dissociation	47.24 (9.99)	41.67 (5.39)	.04

PTSD, posttraumatic stress disorder.

WASI‐IQ, two‐subscale IQ derived from the Wechsler Abbreviated Scales of Intelligence (Wechsler, 1999).

a Wide Range Achievement Test (WRAT 4; Jastak & Wilkinson, 1984).

b Composite score of self‐report and parent rating of Puberty Development Scale (Petersen, Crockett, Richards, & Boxer, 1988).

c Socioeconomic status: Highest level education rated on 6‐point scale from 0 = no formal qualifications to 5 = postgraduate qualification.

d Childhood Trauma Questionnaire (Bernstein & Fink, 1998).

e Trauma Symptom Checklist for Children (Briere, 1996).

### Measures

#### Maltreatment experience

For children referred to SS, maltreatment history, including the estimated severity, onset and duration of maltreatment were provided by the child's social worker or adoptive parent (on the basis of SS records), using an established maltreatment scale (Kaufman, Jones, Stieglitz, Vitulano, & Mannarino, 1994) with an additional rating for intimate partner violence. Severity of each abuse type was rated on a scale from 0 (not present) to 4 (severe). Maltreatment type was rated as follows: neglect *N *=* *18; emotional abuse *N *=* *20; sexual abuse *N *=* *4; physical abuse *N *=* *2; intimate partner violence *N *=* *12. See Appendix S1 for onset, duration and severity by subtype. Additionally, all children completed the Childhood Trauma Questionnaire (CTQ, Bernstein & Fink, 1998; see Appendix S1).

#### Psychiatric symptomatology

The Trauma Symptom Checklist for Children (TSCC; Briere, 1996) was self‐rated to assess posttraumatic symptomatology, depression, anxiety, anger and dissociation symptoms. Average scores in both groups were subclinical threshold (clinical range cut‐off ≥65; see Table 1). Individuals with *T*‐scores within the clinical range were as follows: *N *=* *1 depression, *N *=* *1 anger and *N *=* *2 dissociation in the MT group; *N *=* *1 anxiety in the Control group. The Strengths and Difficulties Questionnaire (Goodman, 1997) was completed by parents and carers to assess broader aspects of functioning (see Appendix S1).

Cognitive ability was assessed using the two subscales of the Wechsler Abbreviated Scales of Intelligence (Wechsler, 1999). Reading ability was assessed with the word reading subscale of the Wide Range Achievement Test (WRAT 4, Jastak & Wilkinson, 1984) to ensure that interpretation of any differences in Stroop performance was not confounded by differences in reading level.

#### Experimental task

Participants underwent an ES task comprising three valence categories following the protocol by Sebastian, Roiser, et al. (2010): rejection‐themed words (e.g. ‘loser’, Rejection condition); inclusion‐themed words (e.g. ‘admired’, Inclusion condition); and neutral words (e.g. ‘cabinet’, Neutral condition). Participants indicated with a button press the ink colour of the stimulus words. Additionally, two classic colour Stroop conditions (CS) were implemented in the same paradigm to formally assess cognitive control (i.e. Incongruent colour words condition and Neutral letter strings condition). The task in the present study aims to elicit incidental processing of rejection‐themed words while children perform a colour‐naming task that ensures they are attending to the stimuli, but which is unlikely to elicit marked behavioural differences across groups. Full details of the stimuli characteristics are available in Appendix S1.

Blocks of each of the five stimulus categories were presented in a permuted design and presented six times over two runs of 7 min. Within each block, 12 words were each presented for 1,500 ms followed by an interstimulus interval of 500 ms. A fixation‐cross appeared after every third block for 15 s. Order of blocks and the order of the words within each block were pseudorandomised. Responses were recorded with button boxes for both hands. RTs, missed trials and error rates were recorded. All participants completed a practice session outside the scanner.

#### fMRI data acquisition

Participants were scanned on a 1.5 Tesla Siemens Avanto MRI scanner (Siemens Medical Systems, Erlangen, Germany) using a 32‐channel head coil and whole brain EPI sequence (parameters: voxel size = 3 × 3 × 3 mm; slices per volume: 35; slice thickness: 2 mm; TR: 2,975 ms; TE: 50 ms; FoV: 192 mm; gap between slices: 1 mm; flip angle: 90°). A magnetisation‐prepared rapid gradient‐echo sequence (MP‐Rage) was used to obtain a high‐resolution structural scan (parameters: 176 slices; slice thickness: 1 mm; gap between slices: 0.5 mm; TE: 2,730 ms; TR: 3.57 ms; FoV: 256 mm; matrix: 256 × 256 mm; voxel size: 1 × 1 × 1 mm). All children's heads were foam padded to minimise head motion.

### Data analyses

Two participants (*N = *1 Maltreated group; *N = *1 Nonmaltreated group) were excluded from analyses because error rates were >2.5 *SD* above the sample mean. Two additional participants (*N = *1 Maltreated group; *N = *1 Nonmaltreated group) were excluded from the behavioural analyses due to a button‐box malfunction but included in fMRI analyses, as their practice files indicated comparable performance. For behavioural data analyses (RT, error and missed trials), please see Appendix S1. Brain images were analysed using SPM8 (www.fil.ion.ucl.ac.uk/spm/software/spm8), implemented in Matlab 2015a (The MathWorks, 2012). The first three volumes were discarded to allow for T1 equilibrium effects. Preprocessing: Each participant's scans were realigned within each run and subsequently across both runs to the first image of run one. Realigned images were coregistered with the individual anatomical T1‐weighted images and subsequently spatially normalised by resampling to a voxel size of 3 × 3 × 3 mm to the standard MNI space (Montreal Neurological Institute). An 8‐mm Gaussian filter was applied to smooth the normalised images and high‐pass filtered at 128 Hz.

Fixed‐effect statistics for each individual were calculated by convolving box‐car functions modelling the five conditions (rejection words; inclusion words; neutral words; incongruent colour words; neutral letter strings) with a canonical hemodynamic response function. To reduce movement‐related artefacts, we additionally included the six motion parameters as regressors and an additional regressor to model images that were corrupted due to head motion >1.5 mm and were replaced by interpolations of adjacent images (<10% of participant's data for *N *=* *9 Nonmaltreated and *N *=* *8 Maltreated; no difference between groups, *p *=* *.18). Second‐level group analyses were conducted using a repeated measures mixed‐effects ANOVA by entering the individual statistical parametric maps containing the parameter estimates of the five conditions as fixed effects and an additional ‘subject factor’ for random effects.

Amygdala ROI‐analyses were small volume corrected (SVC) for multiple comparisons at *p < *.05 using two 8‐mm radius spheres for left and right amygdala, with co‐ordinates based on the protocol by Sebastian, Roiser, et al. (2010). Contrast estimates from the peak voxels of clusters where significant group differences emerged were extracted using the MarsBaR Toolbox (Brett, Anton, Valabregue, & Poline, 2002) implemented in SPM8 and subsequently correlated with PTSD, dissociation and depression subscales of the TSCC (Briere, 1996) in SPSS version 21 (IBM Corp. 2012). For completeness, correlational analyses were also performed with the peak contrast estimates and indices of maltreatment (onset, severity and duration of maltreatment). Whole brain analyses were corrected at cluster level *p *=* *.05, and family‐wise error was determined via Monte‐Carlo simulations with the AFNI programme 3DClustSim (http://afni.nimh.nih.gov/afni; voxel‐wise *p *<* *.005, ke = 75).

## Results

### Behavioural results

There were no main effects of group or group × condition (valence or interference) interactions on the ES or CS task, indicating comparable performance across groups (all *p*s *> *.511). However, as expected, a significant Stroop interference effect was observed for both groups on the CS task (see Appendix S1). Appendix S1 and Table S1 provide details on behavioural performance across conditions.

### fMRI results

#### Emotional Stroop: valence main effects in the Nonmaltreated group

Valence main effects were analysed in the Nonmaltreated group in order to ensure our task conditions elicited activation patterns that were comparable to previous studies. As expected, a main effect of valence in the Nonmaltreated group emerged, with greater BOLD response to rejection words versus neutral words in a fronto‐limbic network, consistent with the pattern seen in typically developing adolescents (Sebastian, Roiser, et al., 2010; see Table S2).

#### Emotional Stroop: valence × group interaction

**Figure 1:**
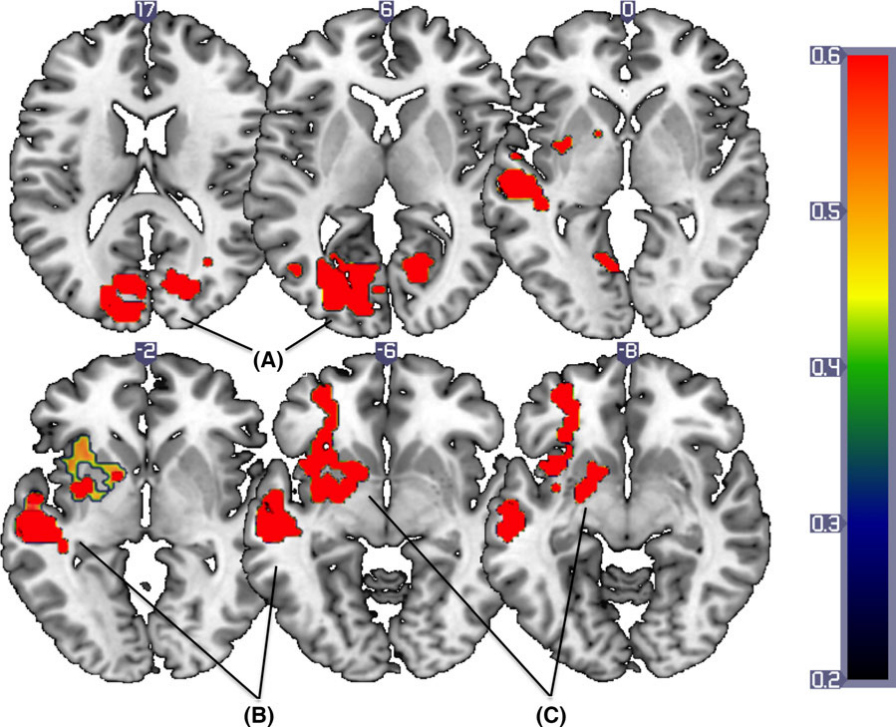
Areas showing attenuated BOLD response in the Maltreated group relative to the Nonmaltreated group in response to the rejection versus neutral words in (A) bilateral visual association cortex (B) left inferior parietal cortex (C) left anterior insula extending into inferior frontal gyrus. Results corrected at *p *=* *.005, ke = 74. Slice numbers reference the MNI coordinate system

A significant valence × group interaction (whole brain level: Rejection vs. Neutral word conditions), indicated that the Maltreated group, relative to their peers, showed reduced activation when processing rejection‐themed words in the left inferior parietal cortex including the STS, bilateral visual association cortex including cuneus as well as the anterior insula extending into the inferior frontal (IFG) and orbitofrontal gyrus (OFC; see Figure 1). The Maltreated group also showed significantly lower neural response in the left amygdala (ROI, *p *=* *.04, *SVC‐corrected*) to rejection versus neutral words. The reverse contrast (Maltreated > Nonmaltreated) for rejection versus neutral words, or the comparison of rejection versus inclusion or inclusion versus neutral yielded no significant group × valence interactions (see Table 2).

**Table 2:** Results of whole brain and region of interest analyses showing group interactions for the emotional and classic Stroop conditions

Brain region	R/L	*x*	*y*	*z*	ke	*Z*
**Rejection words‐Neutral words**						
Nonmaltreated > Maltreated group						
Inferior parietal cortex (STS)	L	−54	−22	−5	98	4.23
	L	−57	−7	−5		3.25
	L	−42	−34	−2		2.85
Visual association cortex	L	−18	−85	13	294	3.69
Cuneus	L	−15	−76	7		3.55
	L	−6	−76	16		3.31
Visual association cortex	R	24	−76	13	105	3.56
Cuneus	R	18	−64	10		3.52
	R	15	−73	13		3.28
Anterior insula	L	−33	−1	−5	113	3.28
Orbitofrontal cortex	L	−33	47	−8		3.23
Thalamus (Pulvinar)	L	−15	5	−5		3.11
Amygdala^a^	L	−24	−4	−8	17	2.94
Maltreated > Nonmaltreated						
		–	–	–	–	
**Rejection‐Incongruent colour words**						
Nonmaltreated > Maltreated group						
Anterior insula	L	−39	14	−8	198	3.82
	L	−33	23	−5		3.49
	L	−39	2	−8		3.43
Maltreated > Nonmaltreated group						
		–	–	–	–	

R/L, right/left; ke, cluster extent.

a Small volume corrected (*p *=* *.04).

### Classic colour Stroop

No significant between‐group differences for congruency (whole brain level: incongruent colour words vs. neutral letter string) were found at the whole brain level. Main effects for the CS conditions are presented in Table S3.

### Isolating the effect of valence by controlling for interference

We wished to isolate the neural activation specific to valence, over and above that elicited by incongruency, by contrasting the Rejection condition (ES) with the Incongruent colour word condition (CS). Main effects are presented in Table S3. A significant valence × group interaction emerged in a large cluster of the left anterior insula; here the Maltreatment group showed significantly reduced BOLD response to Rejection words relative to Incongruent colour words (see Table 2).

### Correlational analyses

**Figure 2:**
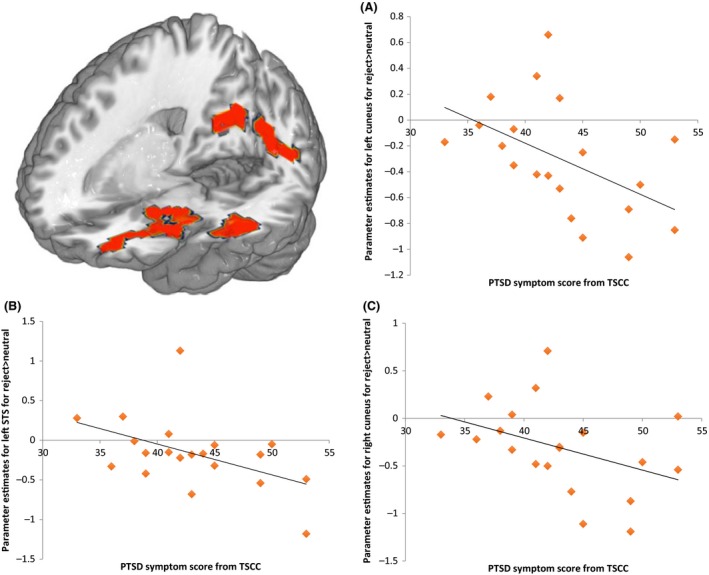
Correlations in the Maltreatment group between posttraumatic stress disorder (PTSD) symptoms (TSCC; Briere, 1996) and parameter estimates for the contrast rejection words > neutral words (ES) for left and right cuneus and STS. ES, emotional Stroop

In relation to PTSD symptoms, significant negative associations were found with bilateral cuneus activation (left: *r*_s_ = −.58, *p *=* *.004; right: *r*_s_ = .52, *p *=* *.017), as well as STS activation (*r*_s_ = −.52, *p *=* *.015); see Figure 2. Additionally, significant negative associations were found between symptoms of dissociation and bilateral cuneus activation (left: *r*_s_ = −.48, *p *=* *.028; right: *r*_s_ = −.457, *p *=* *.037). No significant associations were found in relation to depressive symptoms (*p*s > .105). In the Nonmaltreated group, no significant associations between brain activity and symptoms were found (all *p*s* >* .129). No significant associations were found between the neural activation in maltreatment‐related regions and maltreatment indices (all *p*s > .21).

## Discussion

In the present study, we investigated neural responses to rejection‐themed words in a group of children with documented experiences of maltreatment. Compared to nonmaltreated children, reduced neural response to social rejection‐themed words was observed across a number of brain regions including the left anterior insula, the vlPFC, the amygdala and the STS. These regions are associated with emotion processing, successful inhibition of emotional responses and socioaffective processing more broadly (Etkin & Wager, 2007; Masten et al., 2009), and have been implicated in previous studies of ES interference (Chechko et al., 2013; Sebastian, Roiser, et al., 2010). Our findings suggest that maltreated children are atypical in how they process cues signalling social rejection. In view of the comparable performance across the groups on the classic Stroop task, we were able to eliminate the possibility that differences in general cognitive control processes explained our findings. Additionally, reduced neural responses to rejection‐themed words in the STS and visual association cortex were found to be associated with PTSD symptomatology in the Maltreated group.

These findings indicate that compared to their peers, children who had experienced maltreatment show reduced neural engagement during the incidental processing of stimuli signalling social rejection despite similar behavioural performance. Specifically, whole brain analyses revealed less activity when processing rejection versus neutral words during ES, in regions previously shown to be positively related to adults' and adolescents' distress during exclusion (e.g. vlPFC and anterior insula; Eisenberger, Lieberman, & Williams, 2003; Masten et al., 2009, 2011), while our ROI analysis indicated reduced engagement of the amygdala. Hypoactivations during ES tasks have been observed in patients with affective‐ and trauma‐related disorders. For example, reduced involvement of the vlPFC, parietal and visual cortices have been reported in both patients with major depressive disorder (Chechko et al., 2013) and in those with PTSD (Bremner et al., 2004). In addition, altered responses in visual association areas such as the cuneus and lingual gyrus have been observed in patients with PTSD and dissociative symptoms during ES tasks (Bremner et al., 2004; Shin et al., 1997) and script‐driven imagery symptom provocation paradigms (Hendler et al., 2003). It has been suggested that alterations in the higher order visual association cortices may reflect altered integration of multimodal information and underlie visual and somatosensory symptoms that is relieving the traumatic experience (hyperarousal) or numbing (Lanius, Bluhm, Lanius, & Pain, 2006).

In order to isolate neural response specific to valence, we contrasted the Rejection condition (ES) with the Incongruent colour word condition (CS) which also introduced interference, but without the affective element. This revealed reduced response in the left anterior insula in the Maltreated relative to the Nonmaltreated group. The insula has been implicated in the processing of aversive emotions such as fear (Etkin & Wager, 2007) and is thought to support the interaction between perceived threat signals and bodily states of arousal, including anticipation of pain (Wiech et al., 2010). Like the amygdala, the anterior insula has been reported to show heightened response to facial cues of threat in maltreated individuals (McCrory et al., 2011; Thomaes et al., 2012).

Our finding of attenuated neural response during incidental processing of rejection‐related cues may be interpreted in a number of ways. First, and perhaps most persuasively, it may reflect a pattern of functional avoidance relating to shallower depth of processing in maltreated children during incidental and conscious processing of threat‐related stimuli. It is notable that when maltreated children experience rejection (during the ‘Cyberball’ social rejection paradigm), they are less able to engage neural regions involved in regulation compared to their nonmaltreated peers (Puetz et al., 2014). There are a number of findings from the broader literature, which support the possibility that the pattern of hypoactivation in the current study reflects an avoidant coping style. First, we observed a negative association between PTSD symptomatology and dissociation symptoms and neural response to rejection‐related words. This suggests that those children who most engage in dissociation strategies show the greatest levels of hypoactivation. Second, similar patterns of hypoactivation during ES tasks are seen in patients with PTSD, who by definition are characterised by avoidance (DSM‐5, American Psychiatric Association (APA), 2013). Third, studies of social rejection in other populations have linked deactivation of the anterior insula in particular with maladaptive or avoidant strategies of social engagement, both in adolescents with autism and in adults with an avoidant attachment style (De Wall et al., 2012; Masten et al., 2011). Finally, two previous studies investigating attentional allocation to threat in maltreated children using a dot‐probe paradigm have reported a pattern of attentional bias away from threat when the stimulus can be consciously perceived; this has recently been shown to characterise both males and females equally (Kelly et al., 2015; Pine et al., 2005). However, further experimental studies using, for example, eye tracking during the ES task are needed to provide direct evidence for the avoidant coping style and shallower processing suggested here. In the context of the theory of latent vulnerability, an attenuated neural response to negative social stimuli may reflect an adaptive mechanism of functional avoidance, that is a neural calibration to an adverse home and social environment that is maladaptive in the longer term.

A second interpretation might contend that reduced neural response to rejection‐related cues reflects a developmental delay in cortical maturation associated with the maltreatment experience. For example, studies comparing different age groups on the classic and ES tasks have reported greater Stroop‐related activation in the lateral prefrontal cortex and parieto‐occipital cortices with increasing age (Adleman et al., 2002). Considering the cross‐sectional design of our study, it is not possible to definitively rule out this possibility. In the light of normative behavioural and neural performance on the classic Stroop task in the current study, we consider such a possibility less likely, but longitudinal studies utilising paradigms of social rejection, as well as paradigms that have been designed to specifically interrogate the avoidance strategy hypothesis (e.g. using eye tracking) would arbitrate between these interpretations.

A number of limitations should be noted. First, due to the cross‐sectional design, it was not possible to examine the developmental trajectories of altered processing of rejection in this sample. Future studies employing longitudinal designs could examine if altered neural processing of rejection‐related material predicts future psychopathology in individuals with histories of childhood maltreatment, consistent with the suggestion that this may represent a marker of latent vulnerability. Second, because of our sample size, we were unable to examine the influence of gender, which we know is associated with differential outcomes for boys and girls exposed to early adversity in general (Bos et al., 2011) and maltreatment in particular (Lansford et al., 2002). Third, while we measured symptoms in relation to trauma, anxiety and depression, it is important to note that this did not constitute a general diagnostic measure of psychiatric disorder. Finally, it cannot be fully ruled out that differences in reading strategies influenced our result and future studies should consider using eye tracking in fMRI as a complementary measure.

The present study demonstrates altered neural response during incidental processing of rejection‐related words in children exposed to maltreatment. In the light of the evidence from patients with PTSD and depression, it is conceivable that this neural pattern represents one candidate mechanism indexing latent vulnerability to psychopathology. Longitudinal investigations, however, are needed to establish if such neural calibrations truly index latent vulnerability to subsequent peer problems and mental ill‐health, and whether the neurocognitive mechanism underlying rejection sensitivity is amenable to therapeutic manipulation.

**Key points**

- Childhood maltreatment is associated with heightened perceptual salience of threat‐related facial stimuli, which may represent one candidate mechanism indexing latent vulnerability to future psychiatric disorder. It is unclear, however, if such sensitivity extends to broader cues signalling social threat.
- Using an emotional Stroop task, we found that maltreated children showed reduced activation to rejection‐themed words across circuitry previously implicated in emotion processing and abuse‐related posttraumatic stress disorder.
- One possibility is that this pattern of neural hypoactivation represents a neural calibration to an adverse home environment consistent with avoidant processing.
- While such a response may be adaptive in the short term, an avoidant response style may be maladaptive in the longer term, increasing the risk for psychiatric disorders following exposure to future stressors.

## Supporting information


**Appendix S1.** Measures.
**Table S1.** Behavioural data for the Maltreatment and Nonmaltreatment group for the emotional Stroop conditions and classic colour Stroop conditions.
**Table S2.** Within‐subject results across groups.
**Table S3.** Main effects of congruency (CS) and valence (ES).

## References

[jcpp12595-bib-0001] Adleman, N.E. , Menon, V. , Blasey, C.M. , White, C.D. , Warsofksy, I.S. , Glover, G.H. , & Reiss, A.L. (2002). A developmental fMRI study of the Stroop color‐word task. NeuroImage, 16, 61–75. 11969318 10.1006/nimg.2001.1046

[jcpp12595-bib-0002] APA (2013). DSM‐5 diagnostic and statistical manual of mental disorders (5th edn). Washington, DC: Author.

[jcpp12595-bib-0003] Bernstein, D.P. , & Fink, L. (1998). CTQ Childhood Trauma Questionnaire: A retrospective self‐report. Manual. San Antonio, TX: The Psychological Corporation.

[jcpp12595-bib-0004] Bolger, E. , & Patterson, C.J. (2001). Developmental pathways from child maltreatment to peer rejection. Child Development, 72, 549–568. 11333084 10.1111/1467-8624.00296

[jcpp12595-bib-0005] Bos, K. , Zeanah, C.H. , Fox, N.A. , Drury, S.S. , McLaughlin, K.A. , & Nelson, C.A. (2011). Psychiatric outcomes in young children with a history of institutionalization. Harvard Review of Psychiatry, 19, 15–24. 21250893 10.3109/10673229.2011.549773 PMC3445019

[jcpp12595-bib-0006] Bremner, J.D. , Vermetten, E. , Vythilingam, M. , Afzal, N. , Schmahl, C. , Elzinga, B. , & Charney, D.S. (2004). Neural correlates of the classic color and emotional Stroop in women with abuse‐related posttraumatic stress disorder. Biological Psychiatry, 55, 612–620. 15013830 10.1016/j.biopsych.2003.10.001

[jcpp12595-bib-0007] Brett, M. , Anton, J.L. , Valabregue, R. , & Poline, J.B. (2002). Region of interest analysis using the MarsBar toolbox for SPM 99. NeuroImage, 16, S497.

[jcpp12595-bib-0008] Briere, J. (1996). Trauma Symptom Checklist for Young Children (TSCYC) professional manual. Odessa, FL: Psychological Assessment Resources.

[jcpp12595-bib-0009] Chechko, N. , Augustin, M. , Zvyagintsev, M. , Schneider, F. , Habel, U. , & Kellermann, T. (2013). Brain circuitries involved in emotional interference task in major depression disorder. Journal of Affective Disorders, 149, 136–145. 23394712 10.1016/j.jad.2013.01.013

[jcpp12595-bib-0010] Dalgleish, T. , Taghavi, R. , Neshat‐Doost, H. , Moradi, A. , Canterbury, R. , & Yule, W. (2003). Patterns of processing bias for emotional information across clinical disorders: A comparison of attention, memory, and prospective cognition in children and adolescents with depression, generalized anxiety, and posttraumatic stress disorder. Journal of Clinical Child and Adolescent Psychology, 32, 10–21. 12573928 10.1207/S15374424JCCP3201_02

[jcpp12595-bib-0011] De Ruiter, C. , & Brosschot, J.F. (1994). The emotional Stroop interference effect in anxiety: Attentional bias or cognitive avoidance? Behaviour Research and Therapy, 32, 315–319. 8192630 10.1016/0005-7967(94)90128-7

[jcpp12595-bib-0012] De Wall, C.N. , Masten, C.L. , Powell, C. , Combs, D. , Schurtz, D.R. , & Eisenberger, N.I. (2012). Do neural responses to rejection depend on attachment style? An fMRI study. Social Cognitive and Affective Neuroscience, 7, 184–192. 21467049 10.1093/scan/nsq107 PMC3277372

[jcpp12595-bib-0013] Dresler, T. , Ehlis, A.‐C. , Hindi Attar, C. , Ernst, L.H. , Tupak, S.V. , Hahn, T. , … & Fallgatter, A.J. (2012). Reliability of the emotional Stroop task: An investigation of patients with panic disorder. Journal of Psychiatric Research, 46, 1243–1248. 22770507 10.1016/j.jpsychires.2012.06.006

[jcpp12595-bib-0014] Eisenberger, N.I. , Lieberman, M.D. , & Williams, K.D. (2003). Does rejection hurt? An fMRI study of social exclusion. Science, 302, 290–292. 14551436 10.1126/science.1089134

[jcpp12595-bib-0015] Etkin, A. , & Wager, T. (2007). Functional neuroimaging of anxiety: A meta‐analysis of emotional processing in PTSD, social anxiety disorder, and specific phobia. The American Journal of Psychiatry, 164, 1476–1488. 17898336 10.1176/appi.ajp.2007.07030504 PMC3318959

[jcpp12595-bib-0016] Goodman, R. (1997). The Strengths and Difficulties Questionnaire: A research note. Journal of Child Psychology and Psychiatry, 38, 581–586. 9255702 10.1111/j.1469-7610.1997.tb01545.x

[jcpp12595-bib-0017] Hanson, J.L. , Hariri, A.R. , & Williamson, D.E. (2015). Blunted ventral striatum development in adolescence reflects emotional neglect and predicts depressive symptoms. Biological Psychiatry, 78, 598–605. 26092778 10.1016/j.biopsych.2015.05.010 PMC4593720

[jcpp12595-bib-0018] Hendler, T. , Rotshtein, R. , Yeshurun, Y. , Weizmann, T. , Kahn, I. , Ben‐Bashat, D. , … & Bleich, A. (2003). Sensing the invisible: Differential sensitivity of visual cortex and amygdala to traumatic context. NeuroImage, 19, 587–600. 12880790 10.1016/s1053-8119(03)00141-1

[jcpp12595-bib-0019] IBM Corp. Released (2012). IBM SPSS statistics for Macintosh version 21.0. Armonk, NY: Author.

[jcpp12595-bib-0020] Jastak, S. , & Wilkinson, G.S. (1984). Wide Range Achievement Test‐Revised. Wilmington, DE: Jastak.

[jcpp12595-bib-0021] Kaufman, J. , Jones, B. , Stieglitz, E. , Vitulano, L. , & Mannarino, A.P. (1994). The use of multiple informants to assess children's maltreatment experiences. Journal of Family Violence, 9, 227–248.

[jcpp12595-bib-0022] Kelly, P.A. , Viding, E. , Puetz, V. , Palmer, A.L. , Mechelli, A. , Pingault, J.B. , … & McCrory, E.J. (2015). Sex differences in socio‐emotional functioning, attentional bias and grey matter volume in maltreated children: A multilevel investigation. Development and Psychopathology, 27, 1591–1609. 26535946 10.1017/S0954579415000966

[jcpp12595-bib-0023] Kirke‐Smith, M. , Henry, L. , & Messer, D. (2014). Executive functioning: Developmental consequences on adolescents with histories of maltreatment. The British Journal of Developmental Psychology, 32, 305–319. 24684281 10.1111/bjdp.12041

[jcpp12595-bib-0024] Lanius, R.A. , Bluhm, R. , Lanius, U. , & Pain, C. (2006). A review of neuroimaging studies in PTSD: Heterogeneity of response to symptom provocation. Journal of Psychiatric Research, 40, 709–729. 16214172 10.1016/j.jpsychires.2005.07.007

[jcpp12595-bib-0025] Lansford, J.E. , Dodge, K.A. , Pettit, G.S. , Bates, J.E. , Crozier, J. , & Kaplow, J. (2002). A 12‐year prospective study of the long‐term effects of early child physical maltreatment on psychological, behavioral, and academic problems in adolescence. Archives of Pediatrics and Adolescent Medicine, 156, 824–830. 12144375 10.1001/archpedi.156.8.824 PMC2756659

[jcpp12595-bib-0026] Masten, C.L. , Colich, N.L. , Rudie, J.D. , Bookheimer, S.Y. , Eisenberger, N. , & Dapretto, M. (2011). An fMRI investigation of responses to peer rejection in adolescents with autism spectrum disorders. Developmental Cognitive Neuroscience, 1, 260–270. 22318914 10.1016/j.dcn.2011.01.004 PMC3272329

[jcpp12595-bib-0027] Masten, C.L. , Eisenberger, N.I. , Borofksy, L.A. , Pfeifer, J.H. , McNealy, K. , Mazziotta, J.C. , & Dapretto, M. (2009). Neural correlates of social exclusion during adolescence: Understanding the distress of peer rejection. Social Cognitive and Affective Neuroscience, 4, 143–157. 19470528 10.1093/scan/nsp007 PMC2686232

[jcpp12595-bib-0028] MathWorks (2012). MATLAB and statistics toolbox release. Natick, MA: Author.

[jcpp12595-bib-0500] McCrory, E.J. , De Brito, S.A. , Kelly, P.A. , Bird, G. , Sebastian, C.L. , Mechelli, A. , … & Viding, E. , (2013). Amygdala activation in maltreated children during pre‐attentive emotional processing. The British Journal of Psychiatry, 202, 269–276. 23470285 10.1192/bjp.bp.112.116624

[jcpp12595-bib-0029] McCrory, E.J. , De Brito, S.A. , Sebastian, C.L. , Mechelli, A. , Bird, G. , Kelly, P.A. , & Viding, E. (2011). Heightened neural reactivity to threat in child victims of family violence. Current Biology, 21, 947–948. 10.1016/j.cub.2011.10.015 22153160

[jcpp12595-bib-0030] McCrory, E.J. , & Viding, E. (2015). The theory of latent vulnerability: Reconceptualizing the link between childhood maltreatment and psychiatric disorder. Development and Psychopathology, 27, 493–505. 25997767 10.1017/S0954579415000115

[jcpp12595-bib-0031] O'Dougherty Wright, M. , Crawford, E. , & Del Castillo, D. (2009). Childhood emotional maltreatment and later psychological distress among college students: The mediating role of maladaptive schemas. Child Abuse and Neglect, 33, 59–68. 19167067 10.1016/j.chiabu.2008.12.007

[jcpp12595-bib-0032] Petersen, A.C. , Crockett, L. , Richards, M. , & Boxer, A. (1988). A self‐report measure of pubertal status: Reliability, validity, and initial norms. Journal of Youth and Adolescence, 17, 117–133. 24277579 10.1007/BF01537962

[jcpp12595-bib-0033] Phelps, E.A. , & LeDoux, J.E. (2005). Contributions of the amygdala to emotion processing: From animal models to human behavior. Neuron, 48, 175–187. 16242399 10.1016/j.neuron.2005.09.025

[jcpp12595-bib-0034] Pine, D.S. , Mogg, K. , Bradley, B.P. , Montgomery, L. , Monk, C.S. , McClure, E. , … & Kaufman, J. (2005). Attention bias to threat in maltreated children: Implications for vulnerability to stress‐related psychopathology. American Journal of Psychiatry, 162, 291–296. 15677593 10.1176/appi.ajp.162.2.291

[jcpp12595-bib-0035] Platt, B. , Cohen Kadosh, K. , & Lau, J.Y.F. (2013). The role of peer rejection in adolescent depression. Depression and Anxiety, 30, 809–821. 23596129 10.1002/da.22120

[jcpp12595-bib-0036] Pollak, S.D. , Vardi, S. , Bechner, A.M.P. , & Curtin, J.J. (2005). Physically abused children's regulation of attention in response to hostility. Child Development, 76, 968–977. 16149995 10.1111/j.1467-8624.2005.00890.x

[jcpp12595-bib-0037] Puetz, V.B. , Kohn, N. , Dahmen, B. , Zvyagintsev, M. , Schüppen, A. , Schultz, R.T. , … & Konrad, K. (2014). Neural response to social rejection in children with early separation experiences. Journal of the American Academy of Child and Adolescent Psychiatry, 53, 1328–1337. 25457931 10.1016/j.jaac.2014.09.004

[jcpp12595-bib-0039] Sebastian, C.L. , Roiser, J.P. , Tan, G.C.Y. , Viding, E. , Wood, N.W. , & Blakemore, S.‐J. (2010). Effects of age and MAOA genotype on the neural processing of social rejection. Genes, Brain, and Behaviour, 9, 628–637. 10.1111/j.1601-183X.2010.00596.x 20497231

[jcpp12595-bib-0040] Sebastian, C. , Viding, E. , Williams, K.D. , & Blakemore, S.J. (2010). Social brain development and the affective consequences of ostracism in adolescence. Brain and Cognition, 72, 134–145. 19628323 10.1016/j.bandc.2009.06.008

[jcpp12595-bib-0041] Shields, A.S. , & Cicchetti, D. (2001). Parental maltreatment and emotion dysregulation as risk factors for bullying and victimization in middle childhood. Journal of Clinical and Child Psychology, 30, 349–363. 10.1207/S15374424JCCP3003_7 11501252

[jcpp12595-bib-0042] Shin, L.M. , Kosslyn, S.M. , McNally, R.J. , Alpert, N.M. , Thompson, W.L. , Rauch, S.L. , … & Pitman, R.K. (1997). Visual imagery and perception in posttraumatic stress disorder: A positron emission tomographic investigation. Archives of General Psychiatry, 54, 233–241. 9075464 10.1001/archpsyc.1997.01830150057010

[jcpp12595-bib-0043] Silk, J.S. , Siegle, G.J. , Lee, K.H. , Nelson, E.E. , Stroud, L.R. , & Dahl, R.E. (2014). Increased neural response to peer rejection associated with adolescent depression and pubertal development. Social Cognitive and Affective Neuroscience, 9, 1798–1807. 24273075 10.1093/scan/nst175 PMC4221220

[jcpp12595-bib-0044] Stroop, R.J. (1935). Studies of interference in serial verbal reactions. Journal of Experimental Psychology, XVIII, 643–662.

[jcpp12595-bib-0045] Thomaes, K. , Dorrepaal, E. , Draijer, N. , de Ruiter, M.B. , Elzinga, B.M. , van Balkom, A.J. , … & Veltman, D.J. (2012). Treatment effects on insular and anterior cingulate cortex activation during classic and emotional Stroop interference in child abuse‐related complex post‐traumatic stress disorder. Psychological Medicine, 42, 2337–2349. 22436595 10.1017/S0033291712000499

[jcpp12595-bib-0046] Vachon, D.D. , Krueger, R.F. , Rogosch, F.A. , & Cicchetti, D. (2015). Assessment of the harmful psychiatric and behavioral effects of different forms of child maltreatment. JAMA Psychiatry, 72, 1135–1142. 26465073 10.1001/jamapsychiatry.2015.1792 PMC4699442

[jcpp12595-bib-0047] van Harmelen, A.L. , de Jong, P.J. , Glashouwer, K.A. , Spinhoven, P. , Penninx, B.W.J.H. , & Elzinga, B.M. (2010). Child abuse and negative explicit and automatic self‐associations: The cognitive scars of emotional maltreatment. Behaviour Research and Therapy, 48, 486–494. 20303472 10.1016/j.brat.2010.02.003

[jcpp12595-bib-0048] van Harmelen, A.L. , Hauber, K. , Gunther‐Moor, B. , Spinhoven, P. , Boon, A.E. , Crone, E.A. , & Elzinga, B.M. (2014). Childhood emotional maltreatment severity is associated with dorsal medial prefrontal cortex responsivity to social exclusion in young adults. PLoS ONE, 9, 10–13. 10.1371/journal.pone.0085107 PMC3885678 24416347

[jcpp12595-bib-0049] Wechsler, D. (1999). Wechsler Abbreviated Scale of Intelligence. San Antonio, TX: The Psychological Corporation and Harcourt Brace.

[jcpp12595-bib-0050] Wiech, K. , Lin, C.S. , Brodersen, K.H. , Bingel, U. , Ploner, M. , & Tracey, I. (2010). Anterior insula integrates information about salience into perceptual decisions about pain. Journal of Neuroscience, 30, 16324–16331. 21123578 10.1523/JNEUROSCI.2087-10.2010 PMC6634837

[jcpp12595-bib-0051] Williams, J.M.G. , Mathews, A. , & MacLeod, C. (1996). The emotional Stroop task and psychopathology. Psychological Bulletin, 120, 3–24. 8711015 10.1037/0033-2909.120.1.3

[jcpp12595-bib-0052] Wolfe, D.A. , Scott, K. , Wekerle, C. , & Pittman, A.L. (2001). Child maltreatment: Risk of adjustment problems and dating violence in adolescence. Journal of the American Academy of Child and Adolescent Psychiatry, 40, 282–289. 11288769 10.1097/00004583-200103000-00007

[jcpp12595-bib-0053] Zeanah, C.H. , & Zeanah, P.D. (1989). Intergenerational transmission of maltreatment: Insights from attachment theory and research. Psychiatry, 52, 177–196. 2660176 10.1080/00332747.1989.11024442

