# Supplementary material for: Altered neural response to rejection‐related words in children exposed to maltreatment
Source: J Child Psychol Psychiatry. 2016 Jul 26;57(10):1165–73. doi: 10.1111/jcpp.12595 (PMC5042060; doi:10.1111/jcpp.12595)
Supplement: Supplementary file 1 — Appendix S1. Measures. Table S1. Behavioural data for the Maltreatment and Nonmaltreatment group for the emotional Stroop conditions and classic colour Stroop conditions. Table S2. Within‐subject results across groups. Table S3. Main effects of congruency (CS) and valence (ES). [file JCPP-57-1165-s001.docx]

**Online Appendix Altered neural response to rejection-related words**

**in children exposed to maltreatment** by Puetz, V.B. et al.

**Appendix S1.** Measures

*Psychiatric symptomatology*

*Trauma Symptoms Checklist for Children (TSCC; Briere, 1996).* The Trauma Symptoms Checklist for Children (TSCC; Briere, 1996) was used to assess acute and chronic posttraumatic symptomatology and other symptom clusters. The TSCC is a 44-item self-report measure consisting of five clinical scales (Anger, Depression, Anxiety, Posttraumatic stress and Dissociation). Each item is rated on a four-point scale from *‘never’* to *‘almost all the time’*. Cronbach *α* for the scales ranges from 0.84 to 0.88. TSCC T-scores at or above 65 are considered clinically significant.

*Strength and Difficulties Checklist (SDQ; Goodman, 1997).* The parent-report version of the SDQ (Goodman, 1997) was used to index current social and emotional functioning as well as levels of hyperactivity symptoms and conduct problems. Please see below for total score and subscale scores for both groups.

|  |  | **Maltreated Group (n=21)** | **Non-Maltreated Group (n=19)** |  |
| --- | --- | --- | --- | --- |
|  |  |  |  |  |
|  |  | *Mean (SD)* | *Mean (SD)* | *p* |
| **Strengths & Difficulties Questionnaire (SDQ**) | |  |  |  |
|  | Total Difficulties | 11.95 (7.34) | 5.44 (2.94) | 0.00 |
|  | Emotional Symptoms | 3.05 (2.66) | 1.39 (1.38) | 0.02 |
|  | Conduct Problems | 2.05 (1.83) | 0.61 (0.92) | 0.01 |
|  | Hyperactivity Score | 4.67 (2.58) | 2.5 (1.51) | 0.00 |
|  | Peer Problems | 2.19 (2.14) | 0.94 (1.26) | 0.04 |
|  | Prosocial | 8.14 (2.06) | 8.83 (1.58) | 0.25 |

*Maltreatment ratings (Self-report)*

*Childhood Trauma Questionnaire (CTQ, Bernstein & Fink, 1998).* All children were administered the Childhood Trauma Questionnaire (CTQ, Bernstein & Fink, 1998), a child self-report measure assessing emotional and physical neglect, as well as emotional, physical and sexual abuse, yielding separate scores for each domain as well as a composite overall score; see below.

|  |  | **Maltreated Group (n=21)** | **Non-Maltreated Group (n=19)** |  |
| --- | --- | --- | --- | --- |
|  |  |  |  |  |
|  |  | *Mean (SD)* | *Mean (SD)* | *p* |
| **Type of maltreatment (CTQ score)** | |  |  |  |
|  | Emotional abuse | 7.24 (4.22) | 5.84 (1.68) | 0.19 |
|  | Physical abuse | 6.14 (4.04) | 5.58 (1.50) | 0.57 |
|  | Sexual abuse | 5.00 (0.00) | 5.00 (0.00) | / |
|  | Emotional neglect | 9.62 (4.91) | 6.21 (2.21) | 0.01 |
|  | Physical neglect | 8.05 (3.67) | 5.58 (1.35) | 0.01 |
|  |  |  |  |  |

*Maltreatment ratings (Social Service & Adoptive Parent report)*

|  |  | **Maltreated Group (n=21)** | **Non-Maltreated Group (n=19)** |
| --- | --- | --- | --- |
|  |  |  |  |
| **Measure** |  | *% or Mean (SD)* |  |
|  | |  |  |
| Physical abuse | *n* | 2 (10%) | / |
|  | Severity | 1.00 (0.00) |  |
|  | Onset (years) | 1.54 (2.81) |  |
|  | Duration (years) | 4.00 (1.41) |  |
| Neglect | *n* | 18 (81%) | / |
|  | Severity | 3.22 (0.94) |  |
|  | Onset (years) | 1.45 (3.34) |  |
|  | Duration (years) | 5.95 (4.92) |  |
| Sexual abuse | *n* | 4 (20%) | / |
|  | Severity | 1.50 (1.00) |  |
|  | Onset (years) | 0.93 (1.42) |  |
|  | Duration (years) | 0.25 (0.29) |  |
| Emotional abuse | *n* | 20 (95%) | / |
|  | Severity | 3.10 (0.64) |  |
|  | Onset (years) | 2.01 (3.76) |  |
|  | Duration (years) | 6.06 (4.63) |  |
| Domestic Violence | *n* | 12 (57%) | / |
|  | Severity | 2.17 (1.19) |  |
|  | Onset (years) | 3.19 (3.85) |  |
|  | Duration (years) | 3.47 (3.32) |  |
|  |  |  |  |

Note. Severity of each abuse type (neglect, emotional abuse, sexual abuse, physical abuse, intimate partner violence) was rated on a scale from zero (not present) to four (severe) by the child’s social worker or adoptive parent based on SS records.

*Stimuli*

Stimuli across the ES conditions were matched on frequency (all *p>* .50; Kucera-Francis, 1967), length (all *p>* .13), number of syllables (all *p>* .65) and part of speech (*p>* .55). For all conditions (Rejection, Inclusion, Neutral) normed valence and arousal ratings were taken from the Affective Norms for English Words (Bradley & Lang, 1999). For valence, mean ratings for the rejection, inclusion and neutral words were 1.81, 6.49 and 4.53 respectively (difference between all three conditions: *Ps*< .006). For arousal, mean ratings for the rejection, inclusion and neutral words were 5.60, 4.03 and 3.42 respectively, with both the rejection and inclusion words significantly higher than neutral (*p<*.05) but not differing significantly from each other (*p>*.20).

*Classic Stroop Conditions*

Two additional, classic colour Stroop conditions (CS) were implemented in the same paradigm to formally assess cognitive control following the procedure by Bremner et al. (2004), Thomaes et al. (2012) and Kikuchi et al. (2010). These consisted of colour words written in incongruent colours (e.g. yellow written in green; Incongruent colour word condition) and coloured XXs (Neutral letter string condition). Blocks of these two stimulus categories were presented in a permuted design and presented 6 times over two runs of 7 minutes interspersed pseudo-randomly with the three emotional Stroop conditions. Stimuli were projected onto a screen attached to the front of the scanner in font 8pt on a dark grey background, viewed via a mirror mounted on the head coil and presented using EPrime (Version 2; Schneider, Eschman, & Zuccolotto, 2002).

*Behavioral performance*

Behavioral data (RT, error and missed trials) for the ES were analysed using a 3 x 2 repeated measures ANOVA with valence (Rejection, Inclusion, Neutral words) as the within-subject factor and group (Maltreated vs. Non-maltreated) as the between-subjects factor. Similarly, a 2 x 2 repeated measures ANOVA was conducted for the CS task, with congruency entered as the within-subject factor (Incongruent colour words, Neutral letter strings) and group (Maltreated vs. Non-maltreated) as the between-subjects factor. On the ES task there were no main effects for valence, group or group x valence interactions for RTs, or missed trials (all *Ps*> .83) (see Table S1 in online Appendix for behavioral data by group). Analyses of error rates revealed a significant main effect for valence [*F*(2, 72)=6.90, *p=* .002, *ηp^2^=* .31; mean error Reject=18.55 *±* 1.67; mean error Inclusion=20.66 *±* 1.81; mean error Neutral= 22.79 *±* 1.84]. On the CS task, the expected significant main effect of congruency was observed for RT [*F*(1, 36)=52.81, *p<*.001, *ηp^2^=* .60; mean RT Neutral letter string=763.78 *±* 14.50; mean RT Incongruent words=860.39 *±* 14.57], error [*F*(1, 36)=30.13, *p<* .001, *ηp^2^=*.46; mean error Neutral letter string=19.50 *±* 1.92; mean error Incongruent words=28.32 *±* 2.32] and missed trials [*F*(1,36)=21.59, *p<*.001, *ηp^2^=* .38; mean missed trial Neutral letter string=5.36 *±* 1.04; mean missed trial Incongruent words=10.22 *±* 1.28], with poorer performance on these indices in the Incongruent condition. There were no main effects of group or group x interference interactions indicating comparable performance across groups (all *Ps>*.511).

**Table S1.** Behavioral data for the Maltreatment and Non-Maltreatment group for the emotional Stroop conditions and classic colour Stroop conditions.

|  |  |  |  |  |
| --- | --- | --- | --- | --- |
|  |  | **Maltreated Group (n=20)** |  | **Non-Maltreated Group (n=18)** |
|  |  |  |  |  |
| **RT (millisecond)** | | *Mean (SD)* |  | *Mean (SD)* |
| *Emotional colour Stroop* | | *791 (69)* |  | *800 (101)* |
|  | Rejection words | 793 (72) |  | 804 (105) |
|  | Inclusion words | 791 (57) |  | 801 (97) |
|  | Neutral words | 789 (78) |  | 796 (102) |
|  |  |  |  |  |
| *Classic Stroop* | | 811 (84.5) |  | 813 (96) |
|  | Incongruent colour words | 857 (81) |  | 863 (101) |
|  | Neutral letter string | 765 (88) |  | 763 (91) |
| **Error (%)** |  | *Mean (SD)* |  | *Mean (SD)* |
| *Emotional Stroop* | | 20.4 (11.3) |  | 21.0 (10.4) |
|  | Rejection words | 18.1 (10.6) |  | 19.0 (9.9) |
|  | Inclusion words | 20.5 (11.9) |  | 20.8 (10.2) |
|  | Neutral words | 22.4 (11.5) |  | 23.1 (11.1) |
|  |  |  |  |  |
| *Classic colour Stroop* | | 24.8 (15.9) |  | 23.0 (9.0) |
|  | Incongruent colour words | 29.1 (17.4) |  | 27.5 (9.7) |
|  | Neutral letter string | 20.5 (14.3) |  | 18.5 (8.4) |
| **Missed Trials (%)** | | *Mean (SD)* |  | *Mean (SD)* |
| *Emotional Stroop* | | 5.8 (5.8) |  | 5.2 (5.6) |
|  | Rejection words | 5.2 (5.1) |  | 5.7 (5.3) |
|  | Inclusion words | 6.2 (5.8) |  | 4.8 (5.2) |
|  | Neutral words | 6.1 (6.3) |  | 5.2 (6.4) |
|  |  |  |  |  |
| *Classic colour Stroop* | | 9.1 (9.0) |  | 6.5 (4.1) |
|  | Incongruent colour words | 6.3 (8.1) |  | 4.4 (3.5) |
|  | Neutral letter string | 11.9 (9.9) |  | 8.6 (4.6) |

*Note:* n=1 Maltreatment group and n=1 Non-Maltreatment group are not included in the behavioral analyses due to missing data.

*fMRI results: non-maltreated group*

We first analysed the neural activation patterns in the non-maltreated group in order to ensure our task conditions elicited activation patterns that were comparable to previous studies. These analyses indicated that the Emotional Stroop and the Classical Stroop engaged the fronto-limbic network and a left fronto-parietal network respectively, in line with previous studies of adult and pediatric samples using similar tasks (e.g. Sebastian et al., 2010; Chechko et al., 2013, see online appendix 2 (Table S2) for complete results and coordinates). Compared to Neutral stimuli, Negative-Rejection stimuli elicited greater activity in left superior temporal sulcus (STS), the left vlPFC, specifically the inferior frontal gyrus (IFG) extending into the orbitofrontal gyrus as well as the anterior insula extending into the thalamus. Analyses of activation in predicted regions revealed greater activation in the left amygdala at trend level (*ke*=15, *Z=*2.77, *p*=.06 *SVC-corrected*). This set of brain regions replicates a well-established fronto-limbic network known from the adult and pediatric literature (Sebastian et al., 2010; Chechko et al., 2013), suggesting some comparability of results.

Analyses of neural responses during the classic Stroop yielded greater activation in the Stroop-interference condition as compared to control in a set of left-lateralized fronto-parietal regions implicated in working memory and classic Stroop-tasks (Adult sample: Zysset et al., 2001; Pediatric sample: Adleman et al., 2002), i.e. left dorsolateral prefrontal cortex (dlPFC) extending into IFG, left precuneus and intra-parietal sulcus (IPS) as well as left ventrolateral prefrontal cortex (vlPFC; see Table S2).

*fMRI results: between group differences Incongruent colour words-Neutral letter string (CS)*

No between group differences were found for the contrast Incongruent colour words-Neutral letter string (CS) either for Non-Maltreated >Maltreated group or vice versa. Main effects for this contrast are presented in Table S3.

| **Table S2.** Within-subjects results across groups. | | | | | | |  |  |  |  |
| --- | --- | --- | --- | --- | --- | --- | --- | --- | --- | --- |
|  |  |  |  |  |  |  |  |  |  |  |
| Brain region | **R/L** | **x** | **y** | **z** |  | ***ke*** | | ***t*** | ***Z*** | ***SVC-corrected*** |
|  |  |  |  |  |  |  | |  |  |  |
| ***Contrast: Rejection-Neutral*** |  |  |  |  |  |  | |  |  |  |
| ***Non-maltreated group*** |  |  |  |  |  |  | |  |  |  |
|  |  |  |  |  |  |  | |  |  |  |
| Superior Temporal Sulcus (STS) | L | -57 | -22 | -2 |  | 119 | | 4.21 | 4.11 |  |
|  | L | -42 | -34 | -2 |  |  | | 3.51 | 3.45 |  |
| Inferior frontal gyrus | L | -33 | 47 | -8 |  | 141 | | 3.85 | 3.77 |  |
| Orbitofrontal cortex | L | -48 | 29 | -5 |  |  | | 3.03 | 3 |  |
|  | L | -42 | 38 | -11 |  |  | | 2.79 | 2.76 |  |
| Anterior Insula extenting into Thalamus | L | -27 | 11 | -5 |  | 94 | | 3.60 | 3.54 |  |
|  | L | -24 | -7 | -5 |  |  | | 3.37 | 3.32 |  |
|  | L | -27 | 2 | -5 |  |  | | 3.29 | 3.24 |  |
| Amygdala* | L | -24 | -4 | -8 |  | 15 | | 2.80 | 2.77 | 0.06 |
|  |  |  |  |  |  |  | |  |  |  |
| ***Maltreated group*** |  |  |  |  |  |  | |  |  |  |
|  | -- | -- | -- | -- |  | -- | | -- | -- |  |
|  |  |  |  |  |  |  | |  |  |  |
| Brain region | **R/L** | **x** | **y** | **z** |  | ***ke*** | | ***t*** | ***Z*** | ***SVC-corrected*** |
|  |  |  |  |  |  |  | |  |  |  |
| ***Contrast: Rejection-Acceptance*** |  |  |  |  |  |  | |  |  |  |
| ***Non-maltreated group*** | -- | -- | -- | -- |  | -- | | -- | -- |  |
| ***Maltreated group*** | -- | -- | -- | -- |  | -- | | -- | -- |  |
|  |  |  |  |  |  |  | |  |  |  |
| ***Contrast: Acceptance-Neutral*** |  |  |  |  |  |  | |  |  |  |
| ***Non-maltreated group*** | -- | -- | -- | -- |  | -- | | -- | -- |  |
| ***Maltreated group*** | -- | -- | -- | -- |  | -- | | -- | -- |  |
|  |  |  |  |  |  |  | |  |  |  |
| Brain region | **R/L** | **x** | **y** | **z** |  | ***ke*** | | ***t*** | ***Z*** | ***SVC-corrected*** |
|  |  |  |  |  |  |  | |  |  |  |
| ***Contrast: Incongruent colour words-Neutral letter string (CS)*** |  |  |  |  |  |  | |  |  |  |
|  |  |  |  |  |  |  | |  |  |  |
|  |  |  |  |  |  |  | |  |  |  |
| ***Non-maltreated group*** |  |  |  |  |  |  | |  |  |  |
| Dorsolateral prefrontal cortex (dlPFC) | L | -51 | 14 | 31 |  | 491 | | 5.52 | 5.31 |  |
| Inferior frontal gyrus (IFG) | L | -42 | 20 | 28 |  |  | | 5.42 | 5.22 |  |
|  | L | -51 | 26 | 19 |  |  | | 3.60 | 3.53 |  |
| Intraparietal Sulcus (IPS) | L | -24 | -64 | 43 |  | 544 | | 4.77 | 4.63 |  |
| Praecuneus | L | -36 | -58 | 46 |  |  | | 4.56 | 4.44 |  |
|  | L | -27 | -43 | 43 |  |  | | 3.76 | 3.69 |  |
| Ventrolateral Prefrontal cortex (vlPFC) | L | -51 | 44 | -5 |  | 82 | | 3.68 | 3.61 |  |
|  | L | -33 | 62 | 7 |  |  | | 3.50 | 3.44 |  |
|  | L | -42 | 53 | -2 |  |  | | 3.40 | 3.35 |  |
| Praecuneus | L | -3 | -61 | 43 |  | 109 | | 3.63 | 3.56 |  |
|  | L | -3 | -73 | 49 |  |  | | 3.54 | 3.48 |  |
|  |  |  |  |  |  |  | |  |  |  |
| ***Maltreated group*** |  |  |  |  |  |  | |  |  |  |
|  |  |  |  |  |  |  | |  |  |  |
| Inferior parietal cortex | L | -39 | -46 | 31 |  | 2634 | | 7.4 | 6.93 |  |
|  | L | -33 | -52 | 40 |  |  | | 7.35 | 6.89 |  |
|  | L | -45 | -46 | 40 |  |  | | 6.89 | 6.5 |  |
| Inferior frontal gyrus (IFG) | L | -39 | 8 | 34 |  | 1831 | | 6.43 | 6.11 |  |
|  | L | -42 | 17 | 25 |  |  | | 6.41 | 6.09 |  |
|  | L | -48 | 26 | 19 |  |  | | 5.97 | 5.71 |  |
| Inferior frontal gyrus (IFG) | R | 48 | 14 | 37 |  | 413 | | 4.76 | 4.62 |  |
|  | R | 51 | 29 | 31 |  |  | | 4.14 | 4.05 |  |
|  | R | 42 | 2 | 40 |  |  | | 3.76 | 3.68 |  |
| Ventrolateral Prefrontal cortex (vlPFC) | R | 45 | 50 | -2 |  | 178 | | 4.57 | 4.44 |  |
|  | R | 39 | 59 | 1 |  |  | | 4.21 | 4.11 |  |
|  | R | 48 | 44 | -8 |  |  | | 4.06 | 3.97 |  |
| Middle temporal gyrus (MTG) | L | -54 | -46 | -5 |  | 181 | | 4.23 | 4.13 |  |
|  | L | -48 | -52 | -14 |  |  | | 4.05 | 3.96 |  |
|  | L | -42 | -76 | -8 |  |  | | 4 | 3.91 |  |
|  |  |  |  |  |  |  | |  |  |  |

*Note.* Abbreviations: R/L, Right / Left; ke, cluster extent; SVC-corrected, Small Volume corrected; CS, Classic colour Stroop.

*Region of Interest Analyses

**Table S3.** Main Effects of congruency (CS) and Valence (ES).

| Brain region | **R/L** | **x** | **y** | **z** |  | ***ke*** | ***Z*** |
| --- | --- | --- | --- | --- | --- | --- | --- |
|  |  |  |  |  |  |  |  |
| ***Contrast: Incongruent colour words-Neutral letter string (Main effect CS)*** | | |  |  |  |  |  |
| Dorsolateral Prefrontal Cortex (dlPFC) | L | -42 | 17 | 28 |  | 2589 | 7.28 |
|  | L | -48 | 26 | 19 |  |  | 6.14 |
| Ventrolateral Prefrontal Cortex (vlPFC) | L | -51 | 44 | -5 |  |  | 5.82 |
| Precuneus | L | -24 | -61 | 43 |  | 2506 | 7.23 |
|  | L | -33 | -55 | 43 |  |  | 6.95 |
|  | L | -27 | -70 | 37 |  |  | 6.85 |
| Dorsolateral Prefrontal Cortex (dlPFC) | R | 42 | 56 | -2 |  | 275 | 4.54 |
|  | R | 36 | 59 | 10 |  |  | 3.97 |
| Ventrolateral Prefrontal Cortex (vlPFC) | R | 33 | 41 | 1 |  |  | 3.34 |
| Occipito-Temporal Sulcus | L | -48 | -55 | -14 |  | 202 | 4.21 |
|  | L | -54 | -49 | -8 |  |  | 4.12 |
|  | L | -42 | -76 | -8 |  |  | 3.99 |
|  |  |  |  |  |  |  |  |
|  |  |  |  |  |  |  |  |
|  |  |  |  |  |  |  |  |
| ***Contrast: Rejection words-Incongruent colour words (Main effect ES-CS)*** | | |  |  |  |  |  |
|  |  |  |  |  |  |  |  |
| Temporal Pole /Anterior Insula | L | -45 | 11 | -17 |  | 2157 | 5.73 |
|  | L | -57 | -7 | -14 |  |  | 5.63 |
| Posterior Insula | L | -39 | -19 | 10 |  |  | 4.99 |
| Parahippocampal Gyrus | R | 21 | -34 | -17 |  | 1447 | 5.15 |
| Hippocampus / Amygdala | R | 24 | -16 | -17 |  |  | 5.07 |
| Posterior Insula | R | 45 | -16 | 10 |  |  | 5.06 |
| Ventromedial Prefrontal cortex (vmPFC) | L | -6 | 47 | -8 |  | 455 | 5.04 |
| Subgenual Anterior Cingulate (sgACC) | L | -3 | 20 | -8 |  |  | 4.5 |
|  | R | 6 | 47 | -8 |  |  | 4.4 |
| Visual Association Cortex | R | 9 | -94 | -2 |  | 76 | 3.45 |
|  | R | 21 | -73 | -5 |  |  | 3.15 |
|  | R | 12 | -85 | -2 |  |  | 3.08 |
|  |  |  |  |  |  |  |  |
|  |  |  |  |  |  |  |  |

*Note.* Abbreviations: R/L, Right / Left; ke, cluster extent.
